# A cluster randomised feasibility trial evaluating nutritional interventions in the treatment of malnutrition in care home adult residents

**DOI:** 10.1186/s13063-015-0952-2

**Published:** 2015-09-28

**Authors:** Ruth Stow, Natalie Ives, Christina Smith, Caroline Rick, Alison Rushton

**Affiliations:** Health Research MRes, University of Birmingham, School of Sport, Exercise and Rehabilitation Sciences, Edgbaston, Birmingham, B15 2TT UK; The University of Nottingham, School of Biosciences, Division of Nutritional Sciences, Sutton Bonington campus, Nottingham, LE12 5RD UK; University of Birmingham, Birmingham Clinical Trials Unit, College of Medical and Dental Sciences, Public Health Building, Edgbaston, Birmingham, B15 2TT UK; University College London (UCL), Language & Communication Div of Psychology & Language Sciences, 202d Chandler House, 2 Wakefield Street, London, WC1N 1PF UK; University of Birmingham, School of Sport, Exercise and Rehabilitation Sciences, Edgbaston, Birmingham, B15 2TT UK; Room 30, North Laboratory Building, Sutton Bonington Campus, Leicestershire, LE12 5RD UK

**Keywords:** malnutrition, nutrition support, oral nutritional supplements, sip feeds, care homes, Malnutrition Universal Screening Tool (MUST), elderly, nutritional intervention, nutritional risk

## Abstract

**Background:**

Protein energy malnutrition (PEM) predisposes individuals to disease, delays recovery from illness and reduces quality of life. Care home residents in the United Kingdom are especially vulnerable, with an estimated 30 to 42 % at risk. Evidence for nutritional interventions to address PEM in the care home setting is lacking. Widely used techniques include food-based intervention and/or the use of prescribed oral nutritional supplements. To define outcomes and optimise the design for an adequately powered definitive trial to compare the efficacy of established nutritional interventions in this setting, a cluster randomised feasibility trial with a 6-month intervention was undertaken.

**Methods:**

Care home residents with or at risk of malnutrition were identified across six UK care home sites from September to December 2013. Homes were cluster randomised to standard care (SC), food-based intervention (FB) or oral nutritional supplement intervention (ONS), for 6 months. Key outcomes were trial feasibility and the acceptability of design, allocated interventions and outcome assessments. Anthropometry, dietary intake, healthcare resource usage and participant-reported outcome measures were assessed at baseline and at 3 and 6 months.

**Results:**

All six care homes approached were recruited and retained. Of the 110 residents at risk of malnutrition, 85 % entered the trial, and 68 % completed the 6-month intervention. Pre-specified success criteria for feasibility were met for recruitment and retention, intervention acceptability (resident compliance ≥60 %) and measurement of weight, body mass index (BMI), mid-upper arm circumference and dietary intake (data completeness >80 %). Measurement of handgrip strength and triceps skinfold thickness was not found to be feasible in this population. The 95 % confidence interval (CI) data suggested sensitivity to change in dietary intake for weight, BMI and energy intake between baseline and 3 months when each intervention (FB and ONS) was compared with SC.

**Conclusions:**

A definitive trial comparing the efficacy of nutritional support interventions in increasing weight and BMI in malnourished care home residents can be conducted. However, whilst the design was feasible, this trial has highlighted the lack of clinically and patient-relevant outcome measures that are appropriate for use in this setting for both research and clinical practice. In particular, this trial identified a need for a more simple measure of functional status, which considers the limitations of functional tests in the care home population.

**Trial registration:**

Current Controlled Trials ISRCTN38047922, Date assigned: 22 April 2014.

**Electronic supplementary material:**

The online version of this article (doi:10.1186/s13063-015-0952-2) contains supplementary material, which is available to authorized users.

## Background

Often under-recognised and under-treated, protein energy malnutrition (PEM) develops when energy intake and/or protein intake chronically fail to meet the body’s nutritional requirements [1]. It can affect virtually every function and organ system of the human body [2], predisposing individuals to disease and delaying recovery from illness [3]. In the UK, more than 1 million people over the age of 65 are malnourished or at risk of PEM, and the vast majority (93 %) are residents in a community setting. In 2007, the health and social care costs associated with malnutrition were estimated to exceed £13 billion annually, more than 10 % of the public expenditure on health care [4].

Care homes are arguably home to one of the UK’s most vulnerable populations [5], 30 % to 42 % of whom are estimated to be at risk of malnutrition [6]. PEM significantly impacts the physical and emotional well-being of care home residents and has been linked to increased vulnerability to infection and pressure ulcers, clinical complications, depression and decreased quality of life [7–9]. Evidence for nutritional strategies to address malnutrition in the care home setting is lacking [10]. Widely used dietetic techniques to enhance oral dietary intakes in care homes include food-based intervention (recipe enrichment or fortification of conventional food to increase energy and/or protein density, provision of nourishing snacks and/or fortified drinks) and/or the use of prescribed oral nutritional supplements (ONS) [11], considered to be ‘dietary foods for special medical purposes’ (FSMPs) [12]. The British Dietetic Association (BDA) and The National Prescribing Centre (NPC) advocate improving dietary intake first using fortification of conventional food and secondly by prescribed means [13, 14] However, few trials have evaluated the food-based approach, and whether this type of intervention is able to improve clinical outcomes for malnourished individuals remains unclear [10, 15, 16].

The use of conventional food-based intervention is considered more economical to the National Health Service (NHS) in England than prescribed ONS. In 2011, ONS incurred an annual cost of £105 million, a 10 % increase from 2010. Many General Practitioner (GP) practices identified the significant and increasing costs of ONS at this time, often accompanied by prolonged and inappropriate prescribing. Medicines Management teams subsequently imposed stricter prescribing guidance and encouraged greater use of conventional food-based intervention before or in place of prescribed intervention [17]. However, the development of initiatives to reduce ONS usage prompted concerns about delayed appropriate prescribing [18]. A number of individual components make up complex healthcare interventions such as nutritional support in the care home setting, making it difficult to specify the ‘active ingredient’ of the intervention and to compare intervention variations [19] Whilst there may be potential to improve oral dietary intake in a variety of ways and by using interventions in combination, it is important to establish the effectiveness of both conventional food-based and prescribed ONS interventions to ensure that the most appropriate nutrition support is initiated promptly for vulnerable individuals to minimise further deterioration in nutritional status.

Existing Systematic reviews of nutritional interventions for PEM [20–23] have tended to focus on the effectiveness of ONS compared with placebo or standard care and have been predominantly conducted within the acute setting. A Cochrane meta-analysis, which evaluated the effectiveness of ONS in malnourished older adults [23] reported a small but consistent weight gain with ONS, but the inclusion of primary outcomes of relevance to patients, such as functional measures and quality of life, were lacking, whilst severely malnourished individuals were frequently excluded for ethical reasons. A Cochrane systematic review and meta-analysis was the first to evaluate the impact of dietary advice and/or food-based intervention on PEM [10]. The evidence from the review suggests that dietary advice and intervention for PEM may improve weight and indicators of muscle mass, with or without ONS, but the findings are not specific to the older adult population. Whereas most of the included trials provided information on the duration of the intervention, there was almost no information on the nature, intensity and content of the food-based interventions. The review also highlighted a complete lack of evidence for the effects of food-based intervention on patient-reported outcome measures (PROMs), which are an important determinant of intervention effectiveness.

No systematic review to date has made any specific conclusions regarding nutritional interventions for the treatment of PEM in the care home setting. There is a tendency to avoid research in care homes because of the methodological issues involved [24], and the majority of epidemiological studies either exclude care home residents at the outset or fail to follow up participants when they move into institutional care [25]. The challenges inherent in care home research has led to nutritional intervention trials in this setting that have excluded those with advanced dementia or immobility [16, 26–28], despite these being well-established risk factors for malnutrition. As a result, knowledge of the actual effectiveness of the dietetic interventions for malnutrition that are currently used with this vulnerable population is limited and the clinical applicability of findings to those residents most at risk is often questionable. The lack of evidence to support best practice provides an opportunity to bring new ideas to the field.

To enable the efficacy of these widely used dietetic interventions to be compared within the care home population, an adequately powered randomised controlled trial (RCT) is required. In light of the lack of adequately powered trials with a low risk of bias that have evaluated nutrition support interventions within the care home setting, a feasibility trial was proposed prior to the initiation of a definitive RCT. In this feasibility trial, the research aim was to explore trial design, staff and resident acceptability of the interventions and outcome measures and to provide data to estimate the parameters required to design a definitive RCT.

## Methods

This feasibility trial has been reported with reference to the CONSORT guidelines [29] [Additional file 1].

### Trial design

The trial was conducted in 2013–2014 as a prospective cluster randomised feasibility trial. Three arms were assessed: food-based (FB) intervention, ONS intervention and the standard care (SC) home diet for malnutrition with 6-month intervention duration. Six care home sites were randomised into the three trial arms.

### Trial objectives

The primary objectives of the trial were as follows:

1. To assess how many care homes accepted the invitation to participate in research.
2. To determine whether the eligibility criteria for care home residents were too open or too restrictive by estimating feasible eligibility and recruitment rate.
3. To assess retention of care homes and residents by estimating 3 and 6-month follow-up rates.
4. To investigate the acceptability of nutritional support interventions to malnourished care home residents in terms of compliance and to care home staff in terms of adherence to the intervention schedule.
5. To assess the acceptability and feasibility (and factors influencing this) of the outcome measures as methods to measure efficacy of the interventions within a definitive trial.

The secondary objectives of the trial were as follows:

1. To investigate the completion of screening tools and questionnaires by care home staff.
2. To determine how many malnourished residents were able to participate in PROMs and to complete the questionnaires.
3. To pilot a Healthcare resource usage (HCRU) questionnaire.
4. To measure key outcome domains (for completion rates, missing data, estimates, variances and 95 % confidence intervals for the difference between the intervention arms) for malnourished care home residents, including physical outcome measures and PROMs.
5. To collect and synthesise data, from which the Intracluster Correlation Coefficient (ICC) and sample size of a definitive cluster RCT (CRCT) could be estimated.

### Ethical considerations and research governance

The trial was approved by the West Midlands NHS Local Research Ethics Committee and the Research and Development Department of the Heart of England NHS Foundation Trust prior to commencement. The trial was registered at www.isrctn.com (Current Controlled Trials ISRCTN38047922). The REC approved the consent, randomisation and intervention taking place at the care home level. However, the committee felt that the inclusion of residents lacking capacity in the collection of PROMs could not be justified in accordance with the Mental Capacity Act [31]. They requested that those residents having capacity to complete PROMs, provide individual consent for this part. A joint Data Monitoring Committee/Trial Steering Committee, which included three independent members (a statistician; a dietitian and a member of a patient and public involvement panel), was established prior to trial commencement (August 2013). The DMC/TSC met in January, April and July 2014 to review AEs, mortality and intervention changes. A final meeting was held in November 2014 to discuss the trial findings and inform the evaluation.

### Study settings

The feasibility trial was conducted within the borough of Solihull, West Midlands, in England. Prior to trial commencement, the care and catering staff in 17 care homes providing accommodation for older adults (over 65 years) were receiving regular dietetic input to improve the first-line management of malnutrition. Six, privately owned care homes, selected by purposive sampling to obtain a diverse sample based on type of care provided (residential or nursing/nursing and residential) were invited to take part in the trial. None of the care homes had previously been involved in any research.

### Participants

Recruitment began in September 2013 and concluded in December 2013.

- Care homes (clusters)

Prospective participating care homes were provided with an information sheet and a full explanation of the trial by the Primary Researcher, a registered dietitian with a history of clinical input in all six homes. Each care home was given 1 week to consider participating, after which, the manager was asked to sign a consent form.

- Care home residents

All residents at risk of malnutrition and without a dietetic-led plan in place were considered for eligibility within participating care homes. Care home staff with responsibility for conducting monthly nutritional screening, used the Malnutrition Universal Screening Tool (‘MUST’) to identify those at risk of malnutrition. ‘MUST’ classifies risk as low, medium or high based on body mass index (BMI), history of unintentional weight loss (%) and acute illness effect [11, 30]. ‘MUST’ has been validated for use in adults, has very good-to-excellent inter-observer reliability in care homes (kappa 0.8 to 1.0) and is acceptable to participants and healthcare workers [30]. The REC requested that assessment of eligibility be carried out by non-research staff. Care home staff reviewed the records of those residents identified as being at medium or high risk of malnutrition against the eligibility criteria.

Inclusion criteria for residents were as follows:

1. A score of ‘1’ or higher on the ‘MUST’,
2. Able to eat and drink, and
3. Registered with a Solihull GP and subsequently eligible for the provision of healthcare services provided by the Heart of England NHS foundation Trust (HEFT).

Exclusion criteria for residents were as follows:

1. Receiving (or likely to receive in the next 6 months) enteral tube feeding or parenteral nutrition;
2. Receiving nutritional support in the form of dietetic advice or prescribed ONS;
3. Have a known eating disorder or illness, which requires a therapeutic diet incompatible with fortification and/or supplementation; or
4. On an end-of-life care pathway.

Exclusion criteria for Participant Reported Outcome Measures (PROMs) include the following:

1. Non-native English speaking or
2. Lacking the capacity to consent.

In accordance with the requirements of the approving Research Ethics Committee (REC), residents lacking the capacity to consent were excluded from taking part in PROMs. The decision to exclude non-native English speaking residents, with or without capacity, was based on the Primary Researcher’s existing knowledge of the population group and consideration of the finances available to run the trial. The Primary Researcher estimated that fewer than 5 % of the resident population would be non-native English speaking. This trial was conducted as part of a student MRes project and as such, had no additional funding attached to it. This prohibited the translation of information leaflets into different languages and the hire of an interpreter.

### Individual resident consent for PROMs

Within the care home setting, capacity is assessed by trained care home staff or the GP in accordance with the Mental Capacity Act [31]. Written consent was sought on an individual basis from eligible residents assessed as having functional capacity. Residents were provided with a full explanation of their required participation alongside a Participant Information Sheet. Each resident was asked to sign a consent form

### The interventions

#### Food-based intervention (FB) in addition to standard care (SC)

The content of the FB intervention choices was based on local nutrition support guidelines and national guidance and resources for best practice [11, 32, 33]. These types of interventions are widely recommended by dietitians in clinical practice and are based on ingredients that are commonly used in this setting. Care staff and catering teams in the two homes randomised to FB intervention, received face-to-face instruction from the Primary Researcher to increase the participating resident’s daily nutritional intake by approximately 600 kcal and 20 to 25 g of protein. The choices presented (Table 1) were discussed in relation to the preferences of the resident and the resources and time available at the home. The agreed intervention combination was documented for each resident, at baseline and at 3 months, and recipes were provided. The method of estimating resident food intake being used in all six care homes was the assessment of mealtime servings as a whole, followed by the assignment of a proportion consumed. Staff were asked to continue using this method to record the time of intervention provision and resident intake on the daily food record chart (FRC) as a proportion taken *(All; ¾; ½; ¼; refused).*

**Table 1:** Food-based and ONS intervention composition

Intervention	Recipe	Volume per serving (ml)	Energy content (kcal) per serving	Protein content (g) per serving	Approximate cost per serving
Food-based intervention options					To the care home
Fruit fool	300 ml fruit puree, 150 g custard, 2 tbsp milk powder, 150 ml evaporated milk, 1 tbsp Honey (makes 3)	200	275	7.9	70p
Chocolate mousse	1 sachet instant chocolate dessert, 4 tbsp milk powder, 150 ml double cream, 150 ml full cream milk (makes 2)	150	410	10.95	65p
Milkshake	200 ml full cream milk, 1 tbsp milk powder, 1 tbsp double cream, milkshake powder to taste	200	306	9.8	70p
Fruit smoothie	200 ml full cream milk, 2 tbsp milk powder, 3 tbsp double cream, 1 ripe banana/other fruit, 30 g ice cream	200	306	10	80p
Milky coffee	200 ml full cream milk, 1 tbsp milk powder, 1 heaped tsp of coffee granules, 2 tbsp double cream	200	278	10.6	75p
Malted drink	200 ml full cream milk, 1 tbsp milk powder, 1 tbsp double cream, malted powder (Horlicks/ovaltine or equivalent) to taste	200	304	12	65p
Hot chocolate	200 ml full cream milk, 1 tbsp milk powder, 1 tbsp double cream, drinking chocolate powder to taste	200	306	10.6	65p
ONS intervention options					To the GP practice
Fortisip Bottle (Nutricia Advanced Medical Nutrition)		200	300	12	£2.06
Fortisip Compact (Nutricia Advanced Medical Nutrition)		125	300	12	£2.02
Nutriplen (Nualtra Ltd)		125	300	12	£1.45

The nutritional content of the FB recipes was analysed by the Primary Researcher using the nutritional software package, Diet Plan 6

Fortisip bottle and Fortisip compact are manufactured by Nutricia Advanced Medical Nutrition and were provided by Nutricia for use within the first 3 months of the intervention duration. Nutriplen is manufactured by Nualtra Ltd and was provided by Nualtra for the second 3 months of the intervention duration. The ONS did not incur a cost to the care home GP practice in this trial but would do so within usual care

#### ONS intervention in addition to standard care (SC)

Nursing and/or senior care staff in the two homes allocated to the ONS intervention received instruction by the Primary Researcher to increase the daily nutritional intake of participating residents by approximately 600 kcal and 24 g protein. In accordance with local nutrition support guidelines, the intervention consisted of two liquid ONS (Table 1), provided to residents under the control of a registered dietitian. Staff were asked to record the time of the intervention provision and resident intake on the daily drugs chart as a proportion taken *(All; ¾; ½; ¼; refused).*

The Primary Researcher determined resident compliance with the FB and ONS interventions at 3 and 6 months by calculating average intake from three non-consecutive FRCs or drugs charts. Local nutrition support guidelines advise a minimum first-line ONS prescription of two servings daily. However, the clinical benefits of ONS (unknown for FB intervention) have been seen with one to three servings (300 to 900 kcal) daily [11, 34]. To inform the acceptability of the intervention dose used in this trial, compliance was categorised into ‘non-compliant’, if <50 % (300 kcal) of the advised food/beverage or ONS was consumed daily, compliant with 50 % to 75 % (300 to 450 kcal) of the advised food/beverage/ONS and compliant with 75 % to 100 % (450 to 600 kcal) of the advised food/beverage/ONS. Staff adherence with the intervention schedule was also determined at 3 and 6 months through the collection and review of three non-consecutive FRCs and drugs charts. Adherence was recorded as a percentage of residents that were provided with the agreed intervention type at the agreed frequency of provision.

#### Standard care

All six care homes recruited into the trial had received training and support to provide an SC intervention to residents with or at risk of malnutrition, and all six were considered to be providing adequate standard care. The purpose of a care home SC intervention is to provide a calorie-dense diet [35] through the provision of small, frequent, energy-enriched meals in a ‘family-style’ dining room designed to improve the social ambience around mealtimes. Prompting and assistance is provided by staff where required. SC was provided in all six care homes to ensure that no resident at risk of malnutrition was denied access to first-line treatment [36]. The two care homes allocated to SC only continued to receive visits from the Primary Researcher, but individualised resident plans were not provided.

The Primary Researcher continued to make dietetic visits to all six care homes on a monthly basis. If a resident did not tolerate the allocated intervention (SC, FB or ONS) or experienced a significant decline in nutritional status, a change in nutritional intervention was considered in accordance with local guidelines. Intervention change was recorded for all three trial arms at 3 and 6 months.

### Measures

#### Demographic variables and resident characteristics**:**

Following confirmation of eligibility, care home staff recorded data for each resident on gender, primary diagnosis, capacity, height and diagnosis of dementia and dysphagia. With usual standard care, if measurement of height with a freestanding stadiometer was not possible, the staff in all six homes had been trained to ask residents or relatives for self-reported height or to use ulna length to estimate height from the length of the forearm in accordance with ‘MUST’ [30].

#### Outcome measures

One trial objective was to evaluate feasibility and acceptability of a range of outcome measurements, to establish those most appropriate for a definitive trial. Data completeness of ≥80 % was required for an outcome to be considered for a definitive trial [37]. The primary researcher and the care home staff were responsible for assessment of participating residents (Table 2). To enhance the quality and consistency of staff-assessed outcomes, a training session led by the Primary Researcher was provided, consisting of:

**Table 2:** Assessment Schedule

Measure	Completed by	Assessment Time		
		Baseline	3 months	6 months
Nutritional Intake	Primary Researcher	*√*	*√*	*√*
Weight	Care home staff	*√*	*√*	*√*
BMI	Care home staff	*√*	*√*	*√*
Handgrip Strength	Primary Researcher	*√*	*√*	*√*
MUAC	Primary Researcher	*√*	*√*	*√*
TSF	Primary Researcher	*√*	*√*	*√*
VAS	Participant rated	*√*	*√*	*√*
EQ-5D	Participant rated	*√*	*√*	*√*
CO-OP QoL	Participant rated	*√*	*√*	*√*
Healthcare resource usage	Care home Staff		*√*	*√*

*BMI* body mass index, *MUAC* mid-upper arm circumference, *TSF* tricep skinfold thickness, *VAS* visual analogue scale, *EQ-5D* Euroqol 5 dimensions, *QoL* quality of life

1. Training on the protocols surrounding assessment and recording of outcome measurements
2. Discussion on adverse events and their reporting
3. Mock completion of data collection instruments and form
4. Weight and Body Mass Index (BMI).

Care homes are required to weigh residents on class III approved calibrated scales and to calculate their BMI at least monthly to promote adequate monitoring of nutritional status [38]. Weight and BMI were collected from the care home ‘MUST’ records, along with the number and proportion of residents that could or could not be weighed and the type of scale used. At 3 and 6 months, the primary researcher calculated a repeat ‘MUST’ score for two randomly selected residents per care home and compared the overall score and the scores for each step with that recorded by staff. This enabled competence in calculating BMI and malnutrition risk to be assessed.

- Mid-upper arm circumference (MUAC) and triceps skinfold thickness (TSF)

MUAC, an estimate of subcutaneous fat and arm muscle [39], has been established as a useful indicator of malnutrition risk [40, 41]. TSF is reflective of subcutaneous fat mass and distribution [39] and can be used alongside MUAC to evaluate body composition and to assess nutritional status [42]. The Primary Researcher measured MUAC with a tape measure and TSF with a Slimguide calliper (HaB Essentials), according to standardised procedures. Where possible, the mean of three measurements was recorded to minimise measurement error [43]. The number of residents that refused to have the measurements undertaken, or for whom measurement was not possible, was recorded. MUAC and TSF were used to calculate mid-arm muscle circumference (MAMC), an indicator of protein stores and an estimate of lean muscle mass.

– Handgrip strength
  Handgrip strength an index of general upper extremity strength, is strongly associated with functionalit [44]. Handgrip strength was measured using the Smedley hand held dynamometer (Model 12-0286) on the nondominant arm. Where possible, residents were asked to complete the measurement 3 times on the dominant arm. Due to varying levels of cognitive impairment, it was felt that resident understanding of how to undertake the measure would be poor initially, but improve during the process. The highest achieved measure was therefore recorded. The number of residents that refused to participate, or for whom the measurement was not feasible, was recorded.
– Nutritional Intake Assessment
  To ensure compliance with Outcome 5 (Meeting nutritional needs) of the Essential Standards of Quality and Safety [37], care home staff are required to complete daily food record charts (FRCs) and fluid charts (FCs), to monitor the dietary intake of those with, or at risk of malnutrition. The primary researcher measured and recorded the size/capacity of usual tableware within each care home, at baseline.At each data collection interval, the FRCs and FCs were used to assess the average daily food and fluid intake over three nonconsecutive days. The primary researcher determined daily energy (kcal) and protein (g) intake using the dietary analysis software, Diet Plan 6 (Forestfield Software Ltd, UK). The number of unavailable or incomplete FRCs and FCs was recorded for each care home.

- Healthcare resource usage

Healthcare resource usage data is used alongside health outcomes data to calculate the Incremental Cost-effectiveness Ratio (ICER) [45], defined as the ratio of the difference in cost, to the difference in effectiveness between two intervention strategies. The healthcare resource-usage questionnaire piloted for feasibility by the care home staff in this trial was developed from consideration of existing instruments submitted for use in residential care settings on the ‘MRC Database of Instruments for Resource Use Measurement’ (DIRUM). The questionnaire collected information on hospital admissions, GP call-outs and visits from district nurses, dietitians and Speech and Language Therapists (SaLT).

#### Patient-reported outcome measures (PROMs)

Eligible residents that had provided individual informed consent were provided with the following questionnaires by care home staff.

- Health state: EQ5D-5 L

A core component of economic evaluations in healthcare is the use of preference-based instruments to measure changes in health state. The EuroQol-5D (ED5D-5 L) questionnaire, a standardised, multi-dimensional health state classification [46] consists of a Visual Analogue Scale (VAS), which records self-perceived health status on a scale of 0 to 100 and a descriptive system comprising five dimensions of health (mobility, self-care, usual activities, pain/discomfort and anxiety/depression). The descriptive system can be used to generate a single index value for health state [47], scored in this trial using an algorithm based on a sample from the adult UK population [48].

- Appetite and dietary satisfaction: A Visual Analogue Scale (VAS)

Measured food intake appears to be related to the perceptions of hunger and fullness that can be assessed using a VAS [49, 50]. In this trial a 100 mm VAS was developed to pilot the measurement of each of the following in this setting: ‘hunger’, ‘appetite’, ‘dietary satisfaction’, ‘pleasantness of meals’, ‘pleasantness of snacks’ and ‘pleasantness of drinks’.

- Quality of Life: COOP

Quality of life was assessed using the Dartmouth COOP Quality of life chart, a brief, easy-to-complete questionnaire that is sensitive to subjectively important change [51]. The COOP has been validated in general primary care settings [52] and was piloted in this trial with a care home population.

### Sample size

No formal sample size calculation was performed because the key outcomes were concerned with recruitment, retention and the feasibility and acceptability of the trial [53]. Any investigations of changes in study parameters were exploratory only. Based on the capacity of the selected care homes (29 to 72 residents) and the risk of malnutrition in the UK care home population (30–42 %), it was estimated that between nine (30 % of 29) and 30 (42 % of 72) residents could be considered for receipt of the nutritional intervention within each care home. It was decided that this estimated sample size of *n* = 50 (6 × 9) to *n* = 180 (6 × 30) would provide sufficient data to assess trial feasibility [54].

### Cluster randomisation

A cluster design was chosen primarily to avoid contamination [55] because the care home staff at each site could not be expected to treat participating residents differently.

#### Sequence generation

The random allocation sequence was generated using a computer-generated random number list at the University of Birmingham Clinical Trials Unit. To minimise the time delay between care homes agreeing to participate and implementation of the interventions, care homes were randomised once eligible residents had been identified. This approach is recognised as a means of overcoming delays between recruitment and intervention implementation in cluster trials and was felt to be particularly relevant with the frail, care home population [56].

#### Allocation concealment and mechanism

Concealment of intervention allocation was achieved by giving responsibility for sequence generation and allocation to a statistician independent of the running of the trial. Completing the screening and consent process prior to sequence generation minimised selection bias, thereby ensuring that decisions were not influenced by the assigned intervention.

#### Implementation

The Primary Researcher provided the statistician with the list of care homes that had consented. The statistician stratified and matched clusters according to care type (one nursing and one residential home per pair) and then consecutively numbered all matched pairs. The random allocation sequence was generated, and pairs were assigned to intervention allocation. The statistician notified the primary researcher of the allocations, and each care home was then informed. Staff within each site received training from the primary researcher to support delivery of the allocated intervention and/or a refresher on SC.

### Blinding

Residents were recruited prior to random allocation of care homes to the three arms. As consent was sought at the care home level (aside from PROMs), individual residents were not told of the care home intervention assignment. If a resident questioned the different drink or snack provided, they were told it had been ordered by the dietitian and would be good for them.

Due to the nature of the interventions and the obvious differences between them, it was not possible to blind the staff delivering them. However, nutritional interventions often involve contextual factors that cannot be separated from the intervention itself, such as who delivered the intervention, assistance provided and the setting. It is also acknowledged that the SC trial arm also involved delivery of nutrition support, which may have minimised the differences in resident treatment between trial arms.

This trial had one Primary Researcher, responsible for communicating intervention allocation to participating homes and conducting outcome assessments. It was therefore impossible to blind the primary researcher to the assigned intervention. To minimise bias, the chosen outcome measures were objective and not easily influenced by the observer. In a definitive trial with funding for additional research staff, it should be possible to use observers who assess outcome measures without knowledge of the intervention group.

### Statistical methods

Statistical analyses were performed using IBM SPSS, version 21.

Because effective hypothesis testing requires a powered sample size [53], analysis was limited to descriptive statistics and an exploratory analysis to provide estimates of key parameters and inform the design of a definitive trial. Baseline categorical variables were summarised using proportions, n ( %). All continuous baseline data were tested for normality using Kolmogmorov-Smirnov and were summarised as mean (standard deviation (SD)) or median (interquartile range (IQR)).

Screening logs completed in each care home provided information on the numbers of residents screened using ‘MUST’ and the reasons for not entering the trial. These data, alongside categorical data collected on care home and resident withdrawals, changes to resident intervention, mortality, healthcare resource usage, adverse events and compliance, were summarised (n ( %)) and used to inform aspects of feasibility and acceptability reporting. Continuous outcome measures were summarised as means (SD) or medians (IQR) at 3 and 6 months and mean changes were calculated from baseline to 3 months and 6 months, along with 95 % confidence intervals. The mean difference between ONS and SC and FB and SC were calculated at 3 and 6 months, along with 95 % confidence intervals. This data was used to review the sensitivity of the outcome measures to the change in dietary intake and inform which outcome measures are most appropriate to take forward into a definitive trial.

#### The intracluster correlation coefficient (ICC)

To determine the optimal sample size for a definitive cluster RCT, calculations will be required, which involve the number of clusters, the number of individuals within clusters and the power, significance level and effect size being sought [57]. As the residents in a care home are more likely to be similar, the variability of treatment effects within clusters and the power to detect true differences between intervention arms is reduced [19]. To account for this, an estimate of the magnitude of the intracluster correlation coefficient (ICC), which compares within-group variance to between-group variance [58] will be required for the primary outcome measure to be taken forward. To determine the most appropriate primary outcome for a definitive trial, completion rates and missing data were summarised for all outcome measures, along with estimates and variances.

#### Assessment of feasibility and acceptability

*A priori*, it was specified that the five primary trial objectives would be considered successful if the pre-specified success criteria were met (Table 3).

**Table 3:** Feasibility and acceptability success criteria

Objectives:	Success criteria
Recruitment of care homes	• Recruitment target of six met in the time available (3 months)
Resident eligibility criteria and recruitment	• Favourable difference shown in number at risk of malnutrition and number that were deemed eligible (≤20 % difference)
	• Estimated resident recruitment target of n ≥50 met
Retention of care homes and residents	• Retention of 100 % for care home sites
	• Retention of ≥65 % for residents at 6 months follow-up, accounting for expected high mortality and attrition rates
Intervention acceptability to residents and staff	• Intervention crossover of ≤10 % for each trial arm
	• Given that the clinical benefits of ONS (unknown for FB) are seen with one to three servings (300 to 900 kcal) daily:
	- ≥80 % of residents to be compliant with ≥50 % dietetic-led intervention dose (≥300 to 450 kcal),
	- ≥60 % of residents to be compliant with ≥75 % of the dietetic-led intervention dose (≥450 to 600 kcal)
	• ≥85 % staff adherence to intervention schedule
Feasibility and acceptability of the outcomes piloted	• Data completeness of ≥ 80 %
	• Reported and recorded values were considered complete. Unknown and blank values (due to lack of recording, resident refusal, and inability to measure) were considered missing values.
Objectives:	Success criteria
Recruitment of care homes	• Recruitment target of six met in the time available (3 months)
Resident eligibility criteria and recruitment	• Favourable difference shown in number at risk of malnutrition and number that were deemed eligible (≤20 % difference)
	• Estimated resident recruitment target of n ≥50 met
Retention of care homes and residents	• Retention of 100 % for care home sites
	• Retention of ≥65 % for residents at 6 months follow-up, accounting for expected high mortality and attrition rates
Intervention acceptability to residents and staff	• Intervention crossover of ≤10 % for each trial arm
	• Given that the clinical benefits of ONS (unknown for FB) are seen with one to three servings (300 to 900 kcal) daily:
	- ≥80 % of residents to be compliant with ≥50 % dietetic-led intervention dose (≥300 to 450 kcal),
	- ≥60 % of residents to be compliant with ≥75 % of the dietetic-led intervention dose (≥450 to 600 kcal)
	• ≥85 % staff adherence to intervention schedule
Feasibility and acceptability of the outcomes piloted	• Data completeness of ≥ 80 %
	• Reported and recorded values were considered complete. Unknown and blank values (due to lack of recording, resident refusal, inability to measure) were considered missing values.

### The broader methodological framework of the trial

To address fully the research question and trial objectives, a sequential, explanatory mixed method design was chosen [59], but due to time constraints, it was not realistic to transcribe and analyse the qualitative data within the scope of the MRes project. A mixed methods framework was felt to be necessary to provide a comprehensive analysis of the feasibility and acceptability issues associated with delivering and evaluating nutritional interventions in the care home setting. The use of interviews and focus groups in a qualitative phase would enable the feasibility outcomes to be further explored with the trial participants, ensuring that resident and staff perspectives can be used to inform design and conduct for a definitive trial. The qualitative methodology has not been reported on in this manuscript but will be analysed and reported on separately.

## Results

### Study population: Loss and exclusions

All six care homes approached consented to participate in the trial within the 3-month care home recruitment period (September 2013 to December 2013). All 280 residents living across the six care homes were screened using ‘MUST’, and 110 (39 %) were at medium or high risk of malnutrition. Of the 110 residents, 93 (84.5 %) were eligible to enter the trial and receive the intervention [see Additional file 2]. Reasons for the remaining 17 residents not entering the trial were that they were already receiving dietetic-led nutrition support (*n* = 13), in the hospital (*n* = 2), not registered with a Solihull GP (*n* = 1) and non-English speaking (*n* = 1). Of the 93 residents deemed eligible to enter the trial, only 16 (17 %) were determined by care home staff to have the capacity to consent to PROMs. Written informed consent was obtained from 11 residents, three declined, one was too unwell to be approached and one declined due to family influence. Of the 32 residents in the care homes assigned to SC, two moved out, and 11 died within 6 months. Of the 32 residents in the care homes assigned to FB intervention, two entered end-of-life care, one moved out, and six died. Of the 29 residents in the care homes assigned to ONS intervention, one moved out, one was admitted to the hospital and six died. A total of 63 residents completed the trial by the end of June 2014 and were included in the analyses.

### Baseline data

Demographic data and resident clinical characteristics are shown in Table 4. Included residents were mostly female (82 %), with dementia being the most prominent primary diagnosis (75 %). SC, FB and ONS residents were similar with respect to gender, capacity and diagnosis at baseline. It was considered useful to consider imbalances at baseline within this feasibility trial of few clusters (*n* = 6) to inform the sample size for a definitive trial. There was a noted difference in the proportion of residents at medium and high risk of malnutrition in the care home assigned to the FB intervention (34 % high risk compared to >60 % for the other two arms). The mean values for weight (kg), energy intake (kcal) and fluid intake (ml) were also higher for the FB residents than the residents in the care homes allocated to SC and ONS.

**Table 4:** Demographic and clinical characteristics of residents at baseline (*n* = 93)

	SC (*n* = 32)	FB (*n* = 32)	ONS (*n* = 29)
Gender			
Male	5	6	6
Female	27	26	23
Capacity	5	4	7
Diagnosed dementia	25	25	20
Diagnosed dysphagia	7	4	7
Risk of re-feeding	3	0	4
Malnutrition risk			
High risk	20	11	19
Medium risk	12	20	9
Weight (kg)*	Weighed (*n* = 31)	Weighed (*n* = 32)	Weighed (*n* = 29)
	48.6 (9.1)	55.9 (1.8)	48.2 (10.9)
BMI (kg/m^2^)**	19 (17.0-20.5)	20.1 (18.7-24.8)	18.4 (17.6-21.6)
MAC (cm)	Measured (*n* = 27)	Measured (*n* = 28)	Measured (*n* = 25)
	21.9 (2.7)	23 (2.5)	22 (3.0)
TSF (mm)*	Measured (*n* = 24)	Measured (*n* = 22)	Measured (*n* = 23)
	9.3 (2.8)	13.2 (5.6)	8.4 (3.1)
MAMC (cm)*	*n* = 24	*n* = 22	*n* = 23
	18.9 (2.5)	18.9 (1.7)	18.5 (2.5)
HGD (kg)**	Measured (*n* = 14)	Measured (*n* = 22)	Measured (*n* = 13)
	5.65 (3.9-8.3)	6.9 (4.0-11.5)	5.6 (3.2-10.3)
Energy Intake (kcal)*	Available (*n* = 29)	Available (*n* = 31)	Available (*n* = 27)
	1553 (470)	1916 (496)	1535 (562)
Protein Intake (g)*	Available (*n* = 29)	Available (*n* = 31)	Available (*n* = 27)
	41 (14.6)	78 (22)	54 (20)
Fluid Intake (ml)*	Available (*n* = 27)	Available (*n* = 31)	Available (*n* = 27)
	1109 (237)	1332 (310)	1037 (260)
COOP QoL score**	*n* = 3	*n* = 2	*n* = 6
	5 (5–7)	4 (2–6)	5.5 (4–6)
EQ5D VAS*	*n* = 3	*n* = 2	*n* = 6
	53 (16)	70 (28)	61 (21)
EQ5D index value*	*n* = 3	*n* = 2	*n* = 6
	−0.16 (0.38)	0.15 (0.28)	0.33 (0.33)
Hunger*	*n* = 3	*n* = 2	*n* = 6
	4 (3)	5 (7)	4 (4)
Appetite*	*n* = 3	*n* = 2	*n* = 6
	6 (4)	5.5 (6)	5 (4)
Dietary Satisfaction*	*n* = 3	*n* = 2	*n* = 6
	9 (0.6)	8.5 (2)	8.5 (2)
Pleasantness of meals*	*n* = 3	*n* = 2	*n* = 6
	9 (1)	6 (4)	6 (3)
Pleasantness of snacks*	*n* = 3	*n* = 2	*n* = 6
	7(1.5)	8(1.4)	6(4)
Pleasantness of drinks**	*n* = 3	*n* = 2	*n* = 6
	9(8–10)	9(9–9)	10(8–10)

*SC* standard care, *FB* food-based, *ONS* oral nutritional supplements, *QoL* quality of life, *VAS* visual analogue scale. The number of residents included is indicated for each characteristic *Mean (standard deviation) **Median (interquartile range). EQ-5D index value ranges from −0.59 to 1, with higher scores corresponding to a better health state. COOP score ranges from 1 to 10, with higher scores corresponding to a better QoL

### Outcomes: assessment of feasibility and acceptability

The assessment of the pre-specified success criteria is summarised in Table 5

**Table 5:** Assessment of feasibility and acceptability success criteria

Objectives	Success criteria Met/not met
1. Recruitment of care homes:	Met
	• Six care homes recruited within 3 months
2. Resident eligibility criteria and recruitment	Met
	• 84.5 % of those at risk of malnutrition were eligible for the intervention
	• 93 residents recruited
3. Retention of care homes and residents	Met
	• 100 % of the care homes were retained
	• 68 % of the residents were retained at 6 months
4. Intervention acceptability to residents and staff	Met
	• Intervention crossover of 7.4 % for SC, 4.3 % for ONS arm and 0 % for FB
	• 86 % of residents compliant with ≥50 % of dietetic-led intervention dose at T1 and T2
	• Resident compliance with ≥75 % of the dietetic-led intervention dose at T1 and T2: FB: 78 % and 70 %; ONS: 67 % and 63 %
	• Staff adherence: 100 % for FB at T1 and T2. 100 % for ONS at T1 and 95 % at T2
5. Feasibility and acceptability of the outcomes piloted	Met for
	• Weight, BMI, MUAC, Energy, protein, fluid intake (>80 % data completeness)
	Not met for
	• HGD and TSF (completeness of 54 % and 78 % respectively)

*SC* standard care, *FB* food-based, *ONS* oral nutritional supplements, *BMI* body mass index, *MAC* mid-upper arm circumference, *TSF* tricep skinfold thickness, *HgD* handgrip dynamometer, *T1* Baseline - 3 months, *T2* 3 months - 6 months

#### Retention

All six care homes completed the trial. Of the 93 residents that entered the trial, 67 (72 %) remained at month 3 and 63 (68 %) completed the 6-month intervention. In total, 23 residents died during the trial (25 % mortality), 17 (74 %) of whom were at high risk of malnutrition and 6 (26 %) at medium risk of malnutrition. Of those 23, 87 % died during the first 3 months. Other reasons for loss to follow-up included residents moving out (*n* = 4), entering end-of-life care (*n* = 2) and being admitted to hospital with no planned return (*n* = 1).

#### Acceptability of the allocated interventions

Compliance with the dietetic-led interventions (FB and ONS) was determined at 3 and 6 months. The proportion of fully compliant residents reduced from 74 % at 3 months to 67 % by 6 months, although 86 % of residents during both phases consumed at least half of the provided amount (≥300 kcal) of either FB or ONS intervention. Residents assigned to the FB intervention had greater compliance compared with ONS at both 3 months (78 % versus 67 %) and 6 months (70 % versus 63 %). Reasons for noncompliance were refusal of the intervention (*n* = 4), poor overall intake including the intervention (*n* = 6) and crossover to a different intervention (*n* = 1).

At 3 months, staff adherence to the intervention schedule was 100 % for both ONS and FB intervention. At 6 months, staff adherence with the FB intervention schedule was 100 % and staff adherence to the ONS intervention schedule was 95 %. Drug chart documentation and discussions with staff, revealed that one resident in CH01 was not consistently provided with the agreed upon ONS dose.

#### Acceptability and feasibility of the outcome measures

- Weight

At baseline, seven residents (8 %) were weighed with standing scales, 63 (70 %) with chair scales and 19 (21 %) with hoist scales. Only one resident was unable to be weighed, and that was due to a decline in clinical condition. At 3 months, one resident, weighed by chair scales at baseline, was weighed using hoist scales because of reduced mobility. All other residents in the trial were weighed using the same scales throughout.

- MUAC, TSF and Handgrip strength

Over the three data collection intervals, 88 % of the residents had MUAC measured, 76 % had TSF measured and 54 % had HGD measured. It was not always the same residents that declined or were unable to take part in the measurements. This fluctuation in ability and willingness to participate may be a consequence of the high number of residents with cognitive impairment.

- Nutritional intake

Over the three data collection intervals, 81 % of the FRCs and 87 % of the FCs were available and complete, but despite the training provided to the care homes within usual dietetic practice and prior to trial initiation, there were some limitations to this method of information collection. Whilst the care home staff indicated how much of a meal or snack had been consumed by the resident as a proportion (All, ¾, ½, ¼, refused), there was little to no information on what part of the meal had been eaten, on the recipes used or on specifying whether ingredients had been added to enrich the calorie content. This may have reduced the accuracy of the subsequent dietary analysis and estimation of daily energy, protein and fluid intake.

### Outcomes: Assessment of secondary objectives

- Staff completion of ‘MUST’ tool

At 3 and 6 months, the primary researcher calculated a repeat ‘MUST’ score for two randomly selected residents per care home (*n* = 12). At 3 months, 11 ‘MUST’ scores had been calculated correctly (92 %). Step two (unintentional weight loss) had been calculated over 1 month, rather than 3 to 6 months for one resident. At 6 months, all 12 records (100 %) detailed a correctly calculated score.

- Participant-reported outcome measures (PROMs):

All eligible residents were provided with the questionnaires by care home staff at baseline (*n* = 11), at 3 months (*n* = 8) and at 6 months (*n* = 7), and 100 % of the questionnaires were completed in full. All residents required the care home staff to read the questionnaires to them (due to poor eyesight) and to mark on their responses (due to poor dexterity). Due to the lack of available data, there will be no further analysis reported herein, but resident and staff perceptions of the questionnaires and the perceived ease or difficulty of taking part in PROMs have been explored further within a qualitative phase and will be reported separately.

- Healthcare resource usage questionnaire

The questionnaires were completed in full by care home staff for 100 % of the residents in the trial at both 3 and 6 months. A total of 24 hospital admissions were recorded for 16 residents during the trial. Admissions to A&E accounted for 46 % of the recorded admissions and the majority were for falls (82 %). A total of 117 GP call-outs were recorded for 54 residents during the trial. The most frequently recorded reasons for call-outs were chest examinations (suspected chest infection) (26 %), medication reviews (19 %) and urinary tract infections (15.4 %). District Nurse Visits (63 for 16 residents) were usually scheduled to deliver wound care (37 %) and to check pressure areas (49 %). The number of recorded District Nurse visits was notably higher in the three residential homes, where regular visits were arranged to replace the nursing duties conducted by staff in the care homes providing nursing care. The staff did not directly attribute any admissions, GP or District Nurse visits to a decline in a resident’s nutritional status or to the allocated intervention.

- Data to inform calculation of the intraclass correlation coefficient (ICC) for a definitive trial

In order to conduct a well-designed definitive trial with the aim of comparing efficacy of nutritional interventions, the ICC should be known beforehand to estimate required sample size and statistical power to reduce the chances of type II error [60]. The ICC can be used to determine the increase in variance due to clustering, referred to as the Design Effect (DE), which varies for each outcome measure [61]. Further searching of the existing literature and further piloting within the older adult care home population is required first so as to identify the most appropriate primary outcome measure for a definitive trial and to estimate the population variance of the outcome. The desired power and significance level can be used alongside the anticipated difference between means (effect size), estimated from the literature and pilot work, to calculate the size of the sample that would be required if no clustering was present. The calculated DE can then be used to estimate the necessary inflation of the sample size (compared to that calculated for an individually randomised trial) to take account of the similarities in the clustered data:

### Change in outcomes

To inform which outcome measures are most appropriate to take forwards to a definitive trial, the sensitivity of the outcome measures to the change in oral dietary intake on the introduction of nutritional interventions was assessed by comparing the mean change in outcomes between the intervention arms at 3 and 6 months. Table 6 shows the intervention effects on physical and nutritional outcome measures by T1 (3 months). Where the 95 % confidence interval (CI) of the mean difference (MD) does not cross zero, sensitivity of the outcome measures to a change in oral dietary intake and a difference between the trial arms is suggested. This is observed for weight, BMI and energy intake when each of the intervention arms (net increase in these outcomes) is compared with the SC arm (net decrease) but is not observed when the FB and ONS intervention arms are compared.

**Table 6:** Intervention effects on anthropometric indicators and nutrient intake from baseline to 3 months (*n* = 67)

Outcome	SC (*n* = 19)	FB (*n* = 27)	ONS (*n* = 21)	SC versus FB		SC versus ONS		FB versus ONS	
				(MD)	[95 % CI]	(MD)	[95 % CI]	(MD)	[95 % CI]
Weight change (kg)	Weighed (*n* = 19)	Weighed (*n* = 27)	Weighed (*n* = 21)	−1.9	[−3.6, −0.23]	−2.3	[−4.3, −0.40]	−0.4	[−1.9, 1.1]
	−1.5 (3.3)	0.42 (2.4)	0.82 (2.7)						
BMI change (kg/m^2^)	−0.55 (1.2)	0.16 (1.0)	0.33 (1.2)	−0.7	[−1.4, −0.06]	−0.88	[−1.65, −0.11]	−0.17	[−0.79, 0.44]
MAC change (cm)	Measured(*n* = 13)	Measured (*n* = 24)	Measured(*n* = 18)	−0.77	[−1.7, 0.14]	−0.67	[−1.9, 0.57]		
	−1.06 (1.5)	−0.29 (1.2)	−0.39 (1.8)						
TSF change (mm)	Measured(*n* = 11)	Measured (*n* = 19)	Measured(*n* = 15)	1.15	[−0.26, 2.6]	−0.77	[−2.9, 1.38]		
	0.86 (1.5)	−0.29 (2.0)	1.6 (3.6)						
MAMC change	*n* = 10	*n* = 19	*n* = 16	−1.17	[−2.2, −0.14]	−0.71	[−1.44, 0.02]		
	−1.36 (0.8)	−0.18 (1.5)	−0.65 (0.9)						
HgD change (kg)	Measured (*n* = 7)	Measured (*n* = 17)	Measured (*n* = 6)	0.97	[−1.9, 3.9]	1.62	[−1.34, 4.65]		
	0.16 (2.4)	−0.82 (3.4)	−1.5 (2.5)						
Change in energy intake (kcal)	Available(*n* = 18)	Available (*n* = 27)	Available(*n* = 21)	−380	[−550, −226]	−479	[−697, −263]	−99	[−281, 83]
	−103 (275)	277 (250)	376 (375)						
Change in protein intake (g)	Available(*n* = 18)	Available (*n* = 27)	Available(*n* = 21)	−2.3	[−8.9, 4.3]	−16	[−226, −9.4]		
	0.72 (6.5)	3 (12.9)	16.7 (12.5)						
Change in fluid intake (ml)	Available(*n* = 18)	Available (*n* = 27)	Available(*n* = 21)		Mann–Whitney		Mann–Whitney		
	400 (100–500)*	100 (−20 - 400)*	250 (112.5 - 250)*		U = 164**		U = 110**		

*MD* mean difference, *SC* standard care, *FB* food-based, *ONS* oral nutritional supplements, *BMI* body mass index, *MAC* mid-upper arm circumference, *TSF* tricep skinfold thickness, *MAMC* mid-arm muscle circumference, *HgD* handgrip dynamometer

Normal data is presented as Mean change (standard deviation), otherwise is Median change (interquartile range) (indicated by *). The mean difference (MD) between each dietetic-led intervention arm (FB and ONS) and the SC arm has been calculated for normal data alongside 95 % confidence intervals (CIs), otherwise the Mann–Whitney U Test (indicated by **) has been used. Where sensitivity to change is suggested by the CI, the MD between the FB and ONS arms has then been calculated (final column)

Table 7 shows the intervention effects on physical outcome measures and nutrient intake by T2. There remains a difference in the change in energy intake between each of the intervention arms (net increase in energy intake) and the SC arm (net decrease in energy intake) from baseline to 6 months (T2). As with T1, this was not observed when the FB and ONS intervention arms were compared. The mean change in kcal from baseline to 6 months is less than the added intervention for both of the dietetic-led intervention arms, suggesting that not all of the residents were compliant with the interventions, and some may have reduced their intake of other foods and drinks. The mean change in weight and in BMI over the full 6-month intervention was negative in the SC arm, compared with positive change in each of the dietetic-led intervention arms. However, the 95 % CI crossed zero for each comparison, which suggests that these outcomes were less sensitive to the change in oral dietary intake at T2 compared to T1.

**Table 7:** Intervention effects on anthropometric indicators and nutrient intake from baseline to 6 months (*n* = 63)

Outcome	SC (*n* = 19)	FB (*n* = 23)	ONS (*n* = 21)	SC versus FB		SC versus ONS		FB versus ONS	
				(MD)	[95 % CI]	(MD)	[95 % CI]	(MD)	[95 % CI]
Weight change (kg)	Weighed (*n* = 19)	Weighed (*n* = 23)	Weighed *n* = 21)	−1.4	[−3.6, 0.73]	−1.4	[−3.3, 0.51]		
	−0.57 (3.5)	0.87 (3.4)	0.84 (2.5)						
BMI change (kg/m^2^)	−0.16 (1.3)	0.33 (1.3)	0.34 (1.1)	−0.49	[−1.3, 0.35]	−0.50	[−1.3, 0.27]		
MAC change (cm)	Measured(*n* = 13)	Measured (*n* = 19)	Measured(*n* = 1)	−0.67	[−1.68, 0.34]	−0.82	[−2.2, 0.59]		
	−0.96 (1.6)	−0.29 (1.2)	−0.14 (2.1)						
TSF change (mm)	Measured(*n* = 11)	Measured (*n* = 16)	Measured(*n* = 1)	0.03	[−2.2, 2.26]	−0.97	[−3.8, 1.86]		
	0.68 (2.6)	0.66 (2.9)	1.65 (4.0)						
MAMC change	*n* = 11	*n* = 15	*n* = 14	−0.68	[−1.58, 0.22]	−0.71	[−1.90, 0.33]		
	−1.08 (1.1)	−0.40 (1.1)	−0.29 (1.5)						
HgD change (kg)	Measured (*n* = 6)	Measured (*n* = 11)	Measured(*n* = 6)	2.2	[−0.50, 4.9]	2.2	[−0.61, 4.9]		
	1.8 (2.2)	−0.42 (2.6)	−0.35 (2.1)						
Change in energy intake (kcal)	Available(*n* = 17)	Available (*n* = 23)	Available(*n* = 2)	−255	[−401, −109]	−400	[−577, −223]	−145	[−319,29.1]
	−50.9 (183)	204 (251)	349 (319)						
Change in protein intake (g)	Available(*n* = 17)	Available (*n* = 23)	Available(*n* = 2)		Mann–Whitney		Mann–Whitney		
	1.0 (−3.5 - 4.5)	3.0 (−10 - 6)	9.0 (2–26.5)		U = 175		U = 68.5		
Change in fluid intake (ml)	Available(*n* = 17)	Available (*n* = 23)	Available(*n* = 2)	−79	[−219, 60.9]	−104	[−242, 34.5]		
	120 (223)	199 (210)	224 (196)						

*MD* mean difference, *SC* standard care, *FB* food-based, *ONS* oral nutritional supplements, *BMI* body mass index, *MAC* mid-upper arm circumference, *TSF* tricep skinfold thickness, *MAMC* mid-arm muscle circumference, *HgD* handgrip dynamometer

Normal data is presented as Mean change (standard deviation), otherwise is Median change (interquartile range) (indicated by *). The mean difference (MD) between each dietetic-led intervention arm (FB and ONS) and the SC arm has been calculated for normal data alongside 95 % confidence intervals (CIs), otherwise the Mann–Whitney U Test (indicated by **) has been used. Where sensitivity to change is suggested by the CI, the MD between the FB and ONS arms has then been calculated (final column)

### Harms

Three adverse events were recorded by care home staff during the trial. Details of the adverse events were reduced BMI, placing residents at risk of refeeding syndrome (*n* = 1 SC and *n* =1 FB) and poorly controlled blood glucose levels, reportedly exacerbated by ONS intake (*n* = 1 ONS). One resident with reduced BMI (FB arm) was undergoing investigations led by the GP into possible underlying clinical reasons for ongoing involuntary weight loss. The other two residents were changed to the FB intervention arm.

## Discussion

The feasibility trial design was feasible to undertake in the care home setting. With commitment from staff and management, it was possible to obtain consent from six care homes and to identify 93 residents who were eligible to receive the allocated interventions, meeting the recruitment targets for homes and residents. The success criteria for the retention of care homes and residents throughout the trial were also met; however, resident mortality was high, particularly during the initial 3 months. The three nutritional interventions were considered acceptable to care home residents and staff on the basis of low crossover rates, satisfaction of the success criteria for resident compliance and high staff adherence to the intervention schedules. Of the outcome measures piloted, weight, BMI, MAC and nutritional intake were found to be feasible and acceptable measurements to undertake in this population, meeting the success criteria for data completeness and consideration for use within a definitive trial. Although the trial was not powered to examine intervention outcomes, the direction of effect for weight, BMI and energy intake was in favour of the FB and ONS interventions, highlighting sensitivity to a change in oral dietary intake for these outcome measures.

The following discussion reviews the key findings of the trial in terms of the feasibility and acceptability objectives.

### Recruitment and retention

All six care homes provided written consent and remained in the trial until completion. Prior to trial commencement, the Primary Researcher had established good working relationships with the care home managers and staff within a clinical role. This existing rapport may have been central to successful recruitment and in enabling specific roles to be allocated to staff during the trial process. In a definitive trial, it may be necessary to build in time for researcher visits to potential care homes to establish trust and good communication prior to starting recruitment.

MUST’ screening across the six care homes identified 110 residents (39 %) at risk of malnutrition, a similar percentage to that reported within another care home nutritional intervention trial conducted in Hampshire (37 %) [62]. A total of 84.5 % of those at risk met the eligibility criteria to enter the trial, enabling the recruitment target to be met and supporting the case for a definitive trial in this setting. A large proportion (75 %) of the eligible population presented with a primary diagnosis of dementia. The decision was made to include residents with dementia in order to assess feasibility and acceptability of the trial design with a representative care home population. Assessment of resident characteristics at baseline highlighted imbalances between the intervention arms and indicates a need to recruit a large number of care homes within a definitive trial to increase the likelihood of producing balance across cluster-level covariates. The minimum number of clusters recommended per arm to ensure statistical validity is at least 4 [63].

Despite the fragility of the participating population, it was possible to complete follow-up on 68 % of the residents, thereby achieving the retention target at 6 months. Mortality was the primary reason for residents leaving the trial (23 of 93 in 6 months, 25 %) and appeared to be associated with ‘MUST’ risk category (17 of 23 were high risk, 74 %). Of the 23 residents that passed away, 20 (87 %) had died by month 3, suggesting that at least a proportion may have been nearing end-of-life at recruitment. Although being on an end-of-life care pathway was defined as an exclusion criteria, the restrictions imposed by the approving REC required care home staff to assess resident eligibility and those responsible may have found it challenging to distinguish between residents that may benefit from nutritional intervention and those that were end-stage palliative. In a definitive trial, not subject to the same ethical restrictions, dietetic assessment using a validated method, such as, The Subjective Global Assessment (SGA) [64] could be considered following ‘MUST’ screening to identify where malnutrition is an indication of end-stage disease, as opposed to an indication of inadequate nutritional intake that may respond to intervention.

### Acceptability of the interventions

The small percentage of crossover and adverse events overall (<10 %), suggests that all three interventions were largely acceptable to residents and to staff monitoring nutritional status. The feasibility success criteria for compliance were met, with over 60 % of the residents assigned to FB and to ONS compliant with 450 to 600 kcal/day and over 80 % compliant with at least 300 to 450 kcal/day. Compliance with FB was higher than ONS at 3 and 6 months, perhaps due to the greater variety and flexibility offered by this intervention.

A systematic review conducted in 2012 identified 46 studies of ONS intervention (nine in the care home setting), which included compliance data. Greater compliance was noted with reduced volume, energy-dense ONS (1.5 to 2 kcal/ml) and when a variety of flavours was offered [65], both of which are reported in clinical practice. In this feasibility trial, the ONS were supplied free of charge by two medical nutrition companies, and the bottle size and flavours delivered were dictated by availability. In a fully funded definitive trial, ONS compliance may be improved by specifying use of the 1.5 kcal/ml ONS bottles and by offering residents a choice of flavours. Further qualitative work being undertaken aims to explore the influence of resident and care staff attitudes to FB and ONS intervention on compliance.

### Acceptability and feasibility of the outcome measures and data collection methods

In order to ensure clinical applicability, it was decided that as much data as possible should be collected using methods consistent with usual procedures. Height was collected from ‘MUST’ records for 100 % of residents at baseline. The measurement techniques used had been clearly documented. The majority had been measured using a freestanding stadiometer (66 %), but where this was not possible, estimation by ulna length was the most frequently used alternative measure (31 %). Surrogate methods of height estimation are required for those who are bedridden, confined to a wheelchair or unable to stand straight. The documented use of the technique highlights the importance of training on nutritional assessment prior to research or within clinical practice to increase the efficiency and completeness of documentation.

Measured body weight and calculated BMI were collected from ‘MUST’ documentation at the three data collection intervals. Just one resident had not been weighed at baseline, following a decline in clinical condition and a decision by staff that weighing would be an unnecessary burden. It was possible to retrieve the required information for the remaining 92 residents at baseline and 100 % of the residents remaining at 3 and 6 months. Throughout the trial, only one resident was weighed using different types of scale, justified by the staff, in terms of reduced mobility at the 3-month interval. The success criteria for weight and BMI were achieved, indicating that assessment is acceptable to the majority of residents and is feasible for staff to undertake.

The most widely used method of estimating resident food intake was already being used in all six homes; the assessment of mealtime servings as a whole, following which a proportion consumed is assigned. The majority of FRCs and FCs were available and complete, meeting the success criteria for data completeness. However, limitations relating to the usefulness of the data were observed. Studies that have evaluated the accuracy of care home staff documentation of dietary intake have shown it to be erroneous when compared to independent assessments made by research staff [66, 67]. In this trial, the Primary Researcher did not have capacity to observe resident food and fluid intake and in a definitive trial, intake assessment by research staff would risk losing an important element of testing interventions in the ‘real world’ setting. Improvements in the accuracy of recording could be made by taking photographs of the plate before and after mealtimes. A validation study [66] found this method to be reliable and time-efficient and a possible solution for increasing the accuracy of estimation.

MUAC is included in the ‘MUST’ as an alternative measure for BMI. The MUAC assessment was found to be acceptable to the majority of residents (88 %), enabling the success criteria to be met. A recent cross-sectional care home study, which compared two observers independently assessing MUAC on three occasions over an 8-day period found no systematic differences between observers and concluded that MUAC is acceptable for clinical use in a care home setting [68]. Training and use of a standardised protocol would, however, be essential to ensure accurate and appropriate measurements are undertaken by different observers in a definitive trial.

An average of 76 % of residents in the trial had TSF measured, narrowly missing the success criteria for data completeness. A number of challenges were encountered during measuring, including difficulties encouraging residents to be still, inappropriate positioning of bedridden residents and anxiety in relation to the visual perception of the calliper. This experience suggests that it may prove challenging to accurately perform the measurement in a population with fluctuating capacity and challenging behaviours. Primary problems associated with the TSF measurement include measurement error due to poor technique and substantial differences when measurements are made on the same individual by different observers [69]. These sources of error are likely to be emphasised in the care home population, limiting potential use in a definitive trial.

Handgrip strength is the technique most often recommended for measurement of muscle strength and assessment of muscle function in clinical practice [70]. Only 54 % of residents in this trial were able to undertake the test, and therefore, the success criterion for data completeness was not met. Cognitive impairments made it difficult for residents to understand and follow the instructions, and physical limitations meant some residents were unable to attempt the measure. This is a similar outcome to that noted within other trials conducted in the care home setting where no residents were excluded on the basis of cognitive or physical impairment [71, 72]. These findings suggest that handgrip strength, in its current form, does not enable assessment of muscle strength in the general care home population. There is a requirement for new measurement outcomes for use in a UK care home setting for elderly with functional impairments.

In the absence of any nutrition-specific measures of quality-of-life or health state, this trial assessed the feasibility and acceptability of existing generic tools (EQ5D and COOP), alongside piloting of a VAS to assess appetite and dietary satisfaction. The low numbers recruited significantly reduced the data available to assess feasibility and acceptability, and because the tools were not evaluated with those lacking capacity, the proportion of residents that may be able to respond in a definitive trial remains unknown. The perceived importance and burden of taking part in PROMs, alongside the opinions of staff and residents in relation to whether others could have taken part, have been explored within the qualitative phase of the trial and will be reported on elsewhere.

### Healthcare resource usage

In the absence of a standardised HCRU questionnaire for malnutrition, the questionnaire piloted in this trial was designed to collect information on healthcare professional visits and hospital admissions. Care home staff completed 100 % of the questionnaires at 3 and 6 months, obtaining all of the required data from resident care records and GP visit logs. The questionnaire was able to collect useful data on hospital admissions and professional visits not delivered in-house (dietitian, speech and language therapist), but the data on district nurse and GP visits was likely influenced by the type of care being funded. To enable the questionnaire to be used to inform an economic evaluation of the interventions in a definitive trial, thorough staff training would be required regarding the need to document GP call-outs only. The limitations with the collection of data on nursing visits could be addressed by focusing instead on the reasons for residents requiring nursing care.

The majority of hospital admissions were due to falls, a high proportion of GP call-outs were for assessment or treatment of suspected infections, and the majority of district nurse visits were related to pressure sore care. Neither the care home staff nor the GP attributed any admissions or visits to a resident’s malnourished status, perhaps indicating that awareness of the wide-ranging consequences of malnutrition [18] is limited. It is proposed that the average cost of treating an infection (chest or UTI) or a pressure sore could be used alongside the frequency of occurrence to inform the economic evaluation of the interventions, reducing the reliance on GP or district nurse visit data. Further piloting would be required initially to establish the accessibility and reliability of the necessary data in the care home setting.

### Change in outcomes

For estimated energy (kcal) intake, positive changes favouring the dietetic-led intervention arms were observed at 3 and 6 months, but there was no difference noted between the FB and ONS intervention arms. Increase in total energy intake has been demonstrated in a number of care home malnutrition intervention trials using both FB [15, 73–75] and ONS [27, 62, 73] intervention, which suggests that residents increase their overall energy intake when specific calorie-dense interventions are provided to them on a daily basis. Improved nutritional intake is understood to be a key component in the causal pathway leading to improved clinical outcomes. Change in energy and protein intake should be included within a definitive trial as secondary outcome measures, to examine this relationship further. More accurate means of reporting intake would enable use alongside compliance data, to determine whether habitual intake changes to compensate for the introduction of an intervention.

For recorded weight and BMI, positive changes favouring the dietetic-led interventions were observed when each intervention was compared with SC at 3 and 6 months. Again, there was no observed difference between the FB and ONS arms. An increase in weight and BMI has been reported in a number of care home malnutrition intervention trials using both FB [76, 77] and ONS [26–28] [78, 79] intervention. Whilst such findings suggest these outcomes are sensitive to positive changes in dietary intake, the composition of weight gain is unknown. In terms of delivering functional benefit, a gain of fat mass will not result in improved muscle strength [23]. The limitations associated with weight and BMI as outcomes of clinical relevance has led to calls for more focus on improvement in functional status or quality of life as primary trial outcomes. However, weight and BMI continue to be used in clinical practice as the primary outcome measures for nutrition support interventions.

Negative change was observed for MUAC and MAMC over the 6-month intervention in all three arms, whilst positive change was observed for TSF. Whilst this suggests that weight gain may have been primarily due to an increase in fat mass, observed trends should not be overemphasised due to the feasibility nature of the trial and the lack of complete data for these outcomes. In older adults, low MAMC, a measure of arm muscle area, has been shown to have greater association with mortality than low BMI [80]. Unfortunately, determining MAMC requires TSF measurement and additional calculation, which can hamper practical implementation. The challenges faced within this feasibility trial when attempting to accurately measure TSF suggests that skinfold measurement and therefore, also MAMC, are not appropriate for monitoring short-term changes in body composition, particularly with a population where accurate measurement may be hampered.

### Limitations

The care homes recruited into this trial do not necessarily represent the national care home population. All six had received long-term, regular input from the local dietetic team and were enrolled in a rolling program of staff training. The Primary Researcher had an excellent rapport established with the managers and staff, which may have made it easier to recruit all of the approached sites, to conduct the trial within the required timeframes and to encourage staff to adhere to the protocol. Evaluation of feasibility was undertaken in interested and motivated care homes; thus the transferability of the findings would be dependent upon tailoring to the local context.

Limitations relating to the usefulness of the data from the collected FRCs and FCs were observed, but whilst this may have reduced the accuracy of the estimation of food intake in this trial, it was useful in highlighting that this commonly used method of intake recording may reduce the accuracy of estimated nutritional intake in usual clinical practice. Photographs taken before and after a meal could enable comparisons within a definitive trial to be made in a more accurate manner that does not rely on staff memory. In routine care, the introduction of mealtime photographs alongside staff training and role delegation could be used to estimate nutritional intake and to form important evidence during a CQC inspection.

The restrictions imposed by the approving REC led to limitations in the conduct of the trial. The absence of clinical dietetic assessment following ‘MUST’ screening may have resulted in a number of end-stage palliative care home residents entering the trial and receiving interventions, which were unlikely to deliver nutritional benefit. This lack of expert assessment may have contributed to the high mortality rate during the first 3 months of the trial, reducing the observed effectiveness of the interventions and increasing the risk of attrition bias. Limitations in the design of the trial also increased the risk of performance and detection bias through a lack of double blinding. Whilst the nature of the nutritional interventions under investigation makes it impossible to blind the care home staff to treatment allocation, it should be possible in a fully-funded definitive trial for research staff measuring outcomes and collecting data to be blinded to the allocated intervention.

## Conclusions

The data presented enable several conclusions to be drawn that address the research question and trial objectives. The trial design was feasible to undertake in the care home setting. The established relationships between the Primary Researcher and each of the care home sites may have improved the willingness of staff to follow the trial protocol. Whilst this was explored further during the qualitative phase of the trial (to be reported separately), it is likely that researcher visits to establish trust and to deliver training will need to be accounted for in the timeframe of a definitive trial to aid with successful recruitment of care homes and residents and to culture good relationships with staff teams. This trial demonstrated that a definitive trial comparing the efficacy of FB, ONS and SC interventions in increasing weight and BMI in malnourished care home residents is both feasible and acceptable to undertake. Such a trial could provide useful information as to whether continued NHS expenditure on prescribed ONS is warranted in this setting.

Whilst the design was feasible to undertake, this trial has highlighted a lack of clinically relevant outcome measures, appropriate to this setting, for both research and clinical practice. Many older adults consider functional independence to be more important than the prevention of disease [81, 82], which supports the use of a measure of functional effects as a primary outcome for a future malnutrition intervention trial. This trial identified a need for a more simple measure of functional status, which considers the impediments of functional tests in the care home population. Further qualitative research may be required to explore the nutritional and clinical priorities of those working and residing in the care home setting, alongside the perceived value of nutritional interventions. The results could be interpreted alongside the feasibility outcomes to provide an enhanced understanding of the nutritional care complexities within care homes and to further inform the design of a definitive trial and choice of primary outcome measure.

## Additional files

Additional file 1:
**CONSORT 2010 checklist of information to include when reporting a randomised trial.** This trial has been reported with reference to the CONSORT guidelines. The completed checklist indicates where in the manuscript each item has been addressed. (DOC 217 kb) Additional file 2:
**Consolidated Standards of Reporting Trials CONSORT flow diagram of the conduct of the trial.** Of the 32 residents within the care homes assigned to SC (CH03, CH05), two moved out, and 11 died by 6 months. Of the 32 residents within the care homes assigned to FB intervention (CH04, CH06), two entered end of life care, one moved out, and six died. Of the 29 residents within the care homes assigned to ONS intervention (CH01, CH02), one moved out, one was admitted to hospital and six died. Sixty-three residents completed the trial and were included in the analyses, giving a completion rate of 68 %. (DOCX 84 kb)

## Abbreviations used

PEM: protein energy malnutrition
ONS: oral nutritional supplements
FB: food based
SC: standard care
RCT: randomised controlled trial
GRADE: Grading of Recommendations, Assessment, Development and Evaluation
CQC: care quality commission
REC: Research Ethics Committee
MUST: Malnutrition Universal Screening Tool
BMI: body mass index
MAC: mid-upper arm circumference
TSF: triceps skinfold thickness
MAMC: mid-arm muscle circumference
FRC: food record chart
FC: fluid chart
PROMs: Participant Reported Outcome Measures
QoL: quality of life
VAS: visual analogue scale
HCRU: Healthcare Resource Usage
AE: adverse event
