# Supplementary material for: A cluster randomised feasibility trial evaluating nutritional interventions in the treatment of malnutrition in care home adult residents
Source: Trials. 2015 Sep 28;16:433. doi: 10.1186/s13063-015-0952-2 (PMC4587829; doi:10.1186/s13063-015-0952-2)
Supplement: Additional file 2: — Consolidated Standards of Reporting Trials CONSORT flow diagram of the conduct of the trial. Of the 32 residents within the care homes assigned to SC (CH03, CH05), two moved out, and 11 died by 6 months. Of the 32 residents within the care homes assigned to FB intervention (CH04, CH06), two entered end of life care, one moved out, and six died. Of the 29 residents within the care homes assigned to ONS intervention (CH01, CH02), one moved out, one was admitted to hospital and six died. Sixty-three residents completed the trial and were included in the analyses, giving a completion rate of 68 %. (DOCX 84 kb) [file 13063_2015_952_MOESM2_ESM.docx]

Consolidated Standards of Reporting Trials CONSORT flow diagram of the conduct of the trial

Residents assessed for eligibility to receive the intervention (n=280)

Excluded (n=170) Not at risk of malnutrition

## Recruitment of 6 care homes:

3 Nursing homes (CH01, CH03, CH04)

3 Residential homes

(CH02, CH05, CH06)

Excluded (n=17)

- Receiving nutrition support (n=13)
- In hospital (n=2)
- Not registered with Solihull GP (n=1)
- Non English speaking (n=1)

At risk of malnutrition (n=110)

Eligible residents (n=93)

Randomised care homes (6)

## Allocation (Dec 2013)

1 Nursing home (CH01) and 1 Residential home (CH02) allocated to ONS intervention (n=29)

**Received allocated intervention (n=27)**

**Did not receive intervention (n=2)**

- Died (n=1)
- Moved out (n=1) )

1 Nursing home (CH04) and 1 Residential home (CH06) allocated to FB intervention (n=32)

**Received allocated intervention** **(n=31)**

**Did not receive intervention (n=1)**

- Entered end of life care (n=1)

1 Nursing home (CH03) and 1 Residential home allocated to SC (CH05) (n=32)

**Received allocated intervention (n=32)**

## 3 month follow-Up (March 2014)

**Lost to follow-up** **(n=13)**

- Moved out (n=2)
- Died (n=11)

**Intervention crossover (n=2)**

- to FB (n=1)
- to ONS (n=1)

**Left in the trial (n=19)**

**Lost to follow-up** **(n=6)**

- Hospital (n=1)
- Died (n=5)

**Intervention crossover to FB (n= 1)**

**Left in the trial (n=21)**

**Lost to follow-up** **(n=4)**

- Died (n=3)
- Entered end of life care (n=1)

**Intervention crossover (n=0)**

**Left in the trial (n =27)**

## 6 month follow-Up (June 2014)

**Lost to follow-up** **(n=4)**

- Moved out (n=1)
- Died (n=3)

**Intervention crossover (n=0)**

**Lost to follow-up** **(n=0)**

**Intervention crossover (n=0)**

**Lost to follow-up** **(n=0)**

**Intervention crossover (n=0)**

## Analysis

**Completed trial (n=21)**

**Completed trial (n=23)**

**Completed trial (n=19)**
